# Interstitial pregnancy is one of the most serious and uncommon ectopic pregnancies: Case report

**DOI:** 10.1016/j.ijscr.2022.107195

**Published:** 2022-05-13

**Authors:** Aziz Slaoui, Amine Slaoui, Najia Zeraidi, Amina Lakhdar, Aicha Kharbach, Aziz Baydada

**Affiliations:** a Gynaecology-Obstetrics and Endoscopy Department, Maternity Souissi, University Hospital Center IBN SINA, University Mohammed V, Rabat, Morocco; b Gynaecology-Obstetrics and Endocrinology Department, Maternity Souissi, University Hospital Center IBN SINA, University Mohammed V, Rabat, Morocco; c Urology B Department, Avicenne Hospital, University Hospital Center IBN SINA, University Mohammed V, Rabat, Morocco

**Keywords:** Ectopic pregnancy, Hemorrhagic emergency, Interstitial pregnancy

## Abstract

**Background:**

Ectopic pregnancies are a dreaded and common cause of first-trimester metrorrhagia. They refer to the implantation and development of the embryo outside the uterine cavity. Interstitial localization is uncommon and corresponds to implantation of the embryo in the intramural part of the uterine tube. It has an unforeseen evolution with a risk of cataclysmic hemorrhage by uterine rupture in the absence of early diagnosis and management.

**Case presentation:**

We herein present the uncommon case of a 26-year-old female patient, second gestation, nulliparous, who underwent a pelvic ultrasonography in the emergency department for pelvic pain associated with a two-month amenorrhea. A past history of left salpingectomy for a ruptured tubal ectopic pregnancy 3 years ago was found. Pelvic ultrasound allowed us to detect a ruptured ectopic interstitial pregnancy at 7 weeks of amenorrhea. Significant hemoperitoneum and hemodynamic instability required emergency laparotomy. The condition was confirmed preoperatively and the patient underwent a corneal resection. The postoperative course was uneventful and the patient was discharged on day 4 postoperatively.

**Conclusions:**

The interstitial ectopic pregnancy is an uncommon and life-threatening condition. The importance of early ultrasound detection is of paramount importance to allow conservative treatment with methotrexate injections. Delayed diagnosis requires cornual uterine resection with all the complications that it implies.

## Highlights

• Interstitial ectopic pregnancy is an uncommon and life-threatening condition.
• Conservative management with methotrexate injections can only be proposed after an early diagnosis.
• The importance of early ultrasound screening is emphasized, especially in cases of previous ectopic pregnancies.

## 1 Background

Ectopic pregnancies are a dreaded and common cause of first-trimester metrorrhagia. They refer to the implantation and development of the embryo outside the uterine cavity. Interstitial localization is uncommon and corresponds to implantation of the embryo in the intramural part of the uterine tube [1]. It has an unforeseen evolution with a risk of cataclysmic hemorrhage by uterine rupture in the absence of early diagnosis and management [2]. We herein report the uncommon case of a 26-year-old patient who underwent left salpingectomy for a ruptured tubal ectopic pregnancy, who was diagnosed in our department with a second ruptured ectopic pregnancy, this one interstitial, at 7 weeks of amenorrhea.

## 2 Case presentation

We hereby report the uncommon case of a 26-year-old female patient, second gesture nulliparous with a first ruptured left ectopic tubal pregnancy treated by salpingectomy 3 years ago and a present 3-month pregnancy for which she has not yet benefited from an ultrasonography. She presented to our emergency department with severe pelvic pain radiating along the linea alba and an associated minor black vaginal bleeding. Physical examination revealed hemorrhagic shock with blood pressure at 84 mmHg systolic and 47 mmHg diastolic and tachycardia at 125 beats per minute. The abdomen was rock hard with acute tenderness in the hypogastric region. No metrorrhagia was found in the speculum examination and the vaginal examination found a short posterior cervix. Uterus was enlarged and left lateral vaginal pouch was extremely painful.

**Fig. 1:**
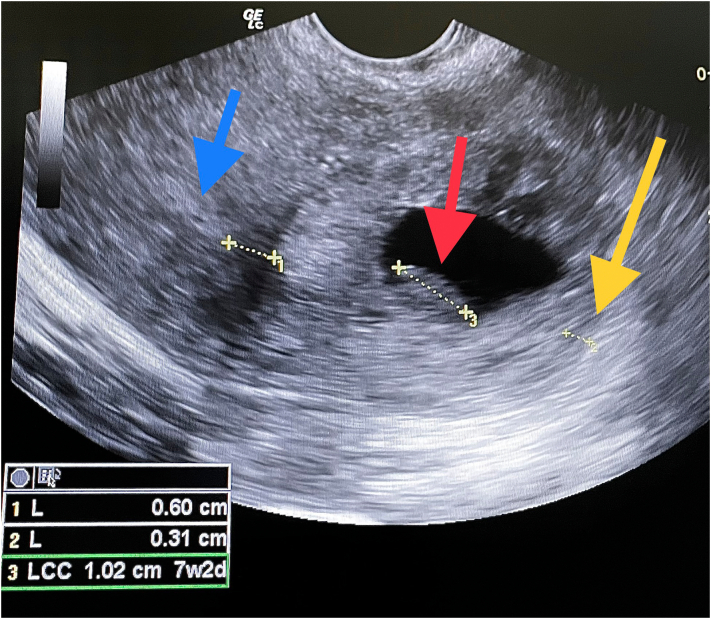
Ultrasound imaging of an interstitial pregnancy. Blue arrow: endometrium; red arrow: embryo; yellow arrow: surrounding myometrium. (For interpretation of the references to colour in this figure legend, the reader is referred to the web version of this article.)

Qualitative urine beta-hCG test came back positive. Pelvic ultrasonography revealed an empty uterus of subnormal size. By rotating the endovaginal probe 30° to the patient's left, the gestational sac appeared with a 10.2 mm craniocaudal length embryo (corresponding to 7 weeks of amenorrhea) without cardiac activity. Gestational sac was found 6 mm lateral to the endometrium and was continuously surrounded by the myometrium with lateral myometrial width of 3 mm (Fig. 1). There was also severe peritoneal effusion in the POD extending around the uterus and through the Morison's pouch. Diagnosis of a ruptured interstitial ectopic pregnancy was highly suspected and the patient was transferred to an operation room without delay.

The patient underwent an exploratory laparotomy under general anesthesia. On opening the peritoneal cavity, a massive hemoperitoneum was found with a mass in the left uterine tubal stump, with breach of myometrial thickness opposite the mass, bleeding profusely. The patient then benefited from a cornual resection and the bleeding was minimized by injection of vasopressin in the periphery of the area being removed. With the uterine margins still hemorrhagic, we added U-shaped hemostasis stitches on top of suturing the margins with a No1 Vicryl until complete closure and satisfactory hemostasis. The uterus, ovaries, and contralateral right tube had a usual appearance. The abdominal cavity was cleaned by saline washing and the abdomen was closed. Operative time was 56 min and total blood loss was evaluated at 200 cm^3^. The patient was transfused with 3 packed red blood cells intraoperatively and 3 packed red blood cells and 2 bags of fresh frozen plasma postoperatively. The postoperative course was uneventful and the patient was discharged home on day 4 postoperatively.

## 3 Discussion

Interstitial, angular and cornual EPs are often classified in the same group, especially in the Anglo-Saxon literature where they are synonymous and represent a single clinical and therapeutic entity [3]. These pregnancies represent about 2% of all EPs [3] and have a mortality rate of 2–2.5%, which is twice that of tubal EPs [4]. However, strictly speaking, interstitial EP develops in the intramural part of the tube. This is a 0.7 mm wide and approximately 1 to 2 cm long duct whose muscular wall is more extensible than the rest of the fallopian tube, sometimes allowing a relatively late development of the pregnancy up to 16 weeks of amenorrhea [5] whereas angular EP develops at the tubal ostium, at the bottom of the uterine horn. The risk of rupture is lower because implantation takes place within the uterine cavity [5]. When it comes to cornual EP, it is defined by the implantation of the sac in the rudimentary horn of a bi-cornual uterus. By extension, this definition includes pregnancies implanted in the horn of a septal uterus or on the stump of a tube in a patient who has undergone salpingectomy [5]. The risk factors are similar to those for tubal EP with the main risk being a history of salpingectomy present in 25 to 40% of cases and found in our patient [6].

In the past, the diagnosis was most often made during exploratory laparotomy for hemorrhagic shock, as in our patient. Indeed, the uterine wall being extensible and very richly vascularized at this site, the rupture of the EP is extremely hemorrhagic. As a consequence, the prevalence of hysterectomy in the case of rupture of an interstitial EP is estimated at 40% and the risk of uterine rupture reaches 20% if the pregnancy continues beyond 12 weeks of gestation [7]. As with classical tubal EP, the diagnosis is based on the synthesis of clinical findings, plasma BHCG positivity and pelvic ultrasound. The clinical presentation is based on the characteristic triad of EP, associating pelvic pain and/or metrorrhagia in a context of amenorrhea with positive BHCG [7]. It may take longer to develop because of local conditions favorable to expansion of the gestational sac, making the clinical signs delayed. However, rupture is frequent and often abrupt [7]. In 1992, Timor-Tritsch et al. [8] described three essential ultrasound criteria for suspecting an interstitial EP: empty uterine cavity, gestational sac eccentric and 10 mm from the endometrium and finally a peripheral myometrial rim less than 5 mm thick. These parameters are very specific (88 to 93%) with low sensitivity (40%) [8]. The ultrasound criteria have remained relatively the same, but more recently some authors recommend the addition of 3D ultrasound, which allows a more precise diagnosis [9]. However, in the event of difficult ultrasound, diagnostic doubt, difficulty in locating a PE and if the clinical condition allows, pelvic MRI remains a very useful complementary examination [10], [11]. Classically, the initial BHCG rate is often higher than for tubal EPs [8]. This can be explained by the greater ease of progression of the horn compared to pregnancy. However, some authors [12] point out that despite technological progress, some interstitial pregnancies are still confused with intrauterine pregnancies that can have cataclysmic consequences.

Until recently, the usual treatment for this type of ectopic pregnancy was homolateral salpingectomy with cornual resection [13], or even hemostasis hysterectomy, which should be avoidable given the advances in imaging [13]. Since the early 1980s, many attempts at conservative medical treatment have been proposed for patients with unruptured interstitial EP [14], [15], [16], [17]. Methotrexate has been used in the literature in a variety of protocols: it has been tested systemically or in situ, as a single injection or in a sequential protocol with repeated injections. Tanaka et al. [15] reported in 1982 the first case of interstitial pregnancy successfully treated with 30 mg MTX IM at D0 and then at D2 and D4. The controversy about the number of injections is not established, although some authors think that repeated doses are more interesting [8], [18]. Recently, the Society of Obstetricians and Gynaecologists of Canada has issued recommendations [19] for the treatment of ectopic interstitial pregnancies. According to them [19], practitioners should first offer conservative medical treatment with multidose and/or local methotrexate in appropriately selected patients. Alternatively, if, as in our patient's case, surgery is required due to hemodynamic criteria or suspected rupture, clinicians may perform laparoscopic cornuostomy or coronal resection, both procedures having comparable outcomes [19].

## 4 Conclusions

The interstitial ectopic pregnancy is an uncommon and life-threatening condition. The importance of early ultrasound detection is of paramount importance to allow conservative treatment with methotrexate injections. Delayed diagnosis requires cornual uterine resection with all the complications that it implies.

This work has been reported in line with the SCARE 2020 criteria [20].

## Abbreviations

EP: ectopic pregnancy
POD: pouch of Douglas
MRI: magnetic resonance imaging

## Ethical approval

Ethics approval has been obtained to proceed with the current study.

## Funding

There are no funding sources to be declared.

## Author contribution

Aziz SLAOUI: study concept and design, data collection, data analysis and interpretation, writing the paper.

Amine SLAOUI: study concept and design, data collection, data analysis and interpretation, writing the paper.

Najia ZERAIDI: study design, data collection, data interpretation, writing the paper.

Amina LAKHDAR: study design, data collection, data interpretation, writing the paper.

Aicha KHARBACH: study design, data collection, data interpretation, writing the paper.

Aziz BAYDADA: study concept, data collection, data analysis, writing the paper.

## Guarantor

The corresponding author is the guarantor of submission.

## Research registration number

Not applicable.

## Consent

Written informed consent was obtained from the patient for publication of this case report and any accompanying images. A copy of the written consent is available for review by the Editor-in-Chief of this journal.

## Availability of data and materials

Supporting material is available if further analysis is needed.

## Declaration of competing interest

The authors declare that they have no competing interests.
